# Bone Morphology in 46 BXD Recombinant Inbred Strains and Femur-Tibia Correlation

**DOI:** 10.1155/2015/728278

**Published:** 2015-02-25

**Authors:** Yueying Zhang, Jinsong Huang, Yan Jiao, Valentin David, Mehmet Kocak, Esra Roan, Denis Di'Angelo, Lu Lu, Karen A. Hasty, Weikuan Gu

**Affiliations:** ^1^Department of Orthopaedic Surgery and Biomedical Engineering, Campbell Clinic, University of Tennessee Health Science Center, Memphis, TN 38163, USA; ^2^Department of Medicine-Nephrology, University of Tennessee Health Science Center, Memphis, TN 38163, USA; ^3^Division of Nephrology, Northwestern University, Chicago, IL 60208, USA; ^4^Department of Preventive Medicine, University of Tennessee Health Science Center, Memphis, TN 38163, USA; ^5^Department of Biomedical Engineering, University of Memphis, Memphis, TN 38152, USA; ^6^Department of Genetics, Genomics and Informatics, University of Tennessee Health Science Center, Memphis, TN 38163, USA

## Abstract

We examined the bone properties of BXD recombinant inbred (RI) mice by analyzing femur and tibia and compared their phenotypes of different compartments. 46 BXD RI mouse strains were analyzed including progenitor C57BL/6J (*n* = 16) and DBA/2J (*n* = 15) and two first filial generations (D2B6F1 and B6D2F1). Strain differences were observed in bone quality and structural properties (*P* < 0.05) in each bone profile (whole bone, cortical bone, or trabecular bone). It is well known that skeletal phenotypes are largely affected by genetic determinants and genders, such as bone mineral density (BMD). While genetics and gender appear expectedly as the major determinants of bone mass and structure, significant correlations were also observed between femur and tibia. More importantly, positive and negative femur-tibia associations indicated that genetic makeup had an influence on skeletal integrity. We conclude that (a) femur-tibia association in bone morphological properties significantly varies from strain to strain, which may be caused by genetic differences among strains, and (b) strainwise variations were seen in bone mass, bone morphology, and bone microarchitecture along with bone structural property.

## 1. Introduction

Osteoporosis is recognized as the most common bone disease in the world. It is characterized with a reduction in bone mass and an alternation of bone microarchitecture, which have been proved to be the major determinants of bone strength. Patients who have osteoporosis are likely to have bone fractures in vertebrae, distal arm, or femoral neck and risk of fracture at many other sites is also increased when bone density is reduced and bone structures are deteriorated, such as tibia [1]. While inbred strains of mice have proven to be useful models for studies of genetic effects on bone structure [2, 3], Turner et al. have showed paradoxical variation between femoral and vertebral strength in inbred strains [4], leading to potential discovery of genetic influence on bone correlations. For example, although C3H mice have significantly stronger femurs compared with B6, their lumbar vertebrae are not stronger, but instead they are more brittle. This result indicated that the genes contributing to improved femoral strength have no effect or even negative effect on trabecular bone structure in the spine. However, there has been no study demonstrating the predictability between long bones.

Additionally, previous genetic study on correlation of bone quality of long bones has been limited to classic inbred strains, such as C57BL/6J (B6) and DBA/2J (D2), where few successes for genes contributing to complex, multigenic traits have been achieved [5, 6]. Comparison using mice in F2 population seems exceedingly complex because of the genomic heterozygosity of the F2 population. In the current study, we evaluated the bone mass and microstructure of femurs and tibias in BXD RI mouse strains. Recombinant inbred (RI) strains were created by intercrossing two inbred lines (often classical lines) and then breeding them to homozygosity through more than 20 generations of sibling mating, meaning mating brothers and sisters from the same strain. BXD RI strains were derived by intercrossing B6 and D2. The resulting F1 generation was inbred to produce F2 and subsequent generations. Then, brother and sister pairs in F5 were used to produce offspring BXD strains by inbreeding over 20 generations to ensure that BXD strains were all homozygous. Some BXD strains became distinct and thus were not included in the study (e.g., BXD47, BXD58, and BXD72) [7].

We hypothesized that associations between long bone morphologies are affected by heritable components. To test this hypothesis, we quantified femoral and tibial bone structure and density in 46 BXD RI strains and the progenitor B6 and D2 strains. If genetic control impacted femur-tibia correlation, a variety of associations could be revealed across strains.

## 2. Materials and Methods

### 2.1. Animal

All mice used in the experiments were sacrificed using a protocol approved by the Memphis Veterans Affairs (VA) Medical Center (appendix). Strict breeding environment provides a means to circumvent complicating environmental factors. These mice were originally obtained from the Center for Neuroscience, Department of Anatomy and Neurobiology, University of Tennessee Health Science Center (855 Monroe Avenue Memphis, Tennessee). The male and female mice were from two progenitor strains C57BL/6J (*n* = 16) and DBA/2J (*n* = 15) and we phenotyped 47 BXD recombinant inbred (RI) strains (from 9.57 to 13.57 weeks old). There were 46 BXD recombinant inbred strains that had sufficient number of animals (*n* ≥ 3 for each strain) for testing. There were 41 strains with male and female animals, 5 strains only with males, and 1 strain only with females. D2B6F1 (*n* = 9) were derived from an intercross of the progenitor strains (female DBA/2J and male C57BL/6) and B6D2F1 (*n* = 7) were derived from an intercross between female C57BL/6 and male DBA/2J. In total, 358 mice were collected for sacrifice.

After all, one femur and one tibia were collected free of soft tissue observed by naked eye from each mouse. A total of 696 mice long bones were collected (355 femurs and 341 tibias) and 611 of them were included in the study (337 femurs and 274 tibias) to ensure a sufficient number of samples (*n* ≥ 3) for each strain. Bones were harvested postmortem and cleared off of the surrounding connective tissue as could be seen by eyes for contrast enhancement in *μ*CT and energy expenditure reduction in mechanical test. The femoral heads and necks are retained on the femurs, while the fibulas were removed from tibia.

### 2.2. Specimens Handling

Bone specimens that underwent X-ray imaging were required to be preserved and stored with special care. Ethanol preserves protein (bone marrow), bone mineralization, and hydration. In this study, 70% ethanol was used to preserve bone specimens for best morphological examination (maintaining protein structure) whereas formalin could be best used for bone histology.

### 2.3. High-Resolution *μ*CT

In order to quantitatively assess the structural changes with genetic variation, morphometric and architectural indices were determined from the microtomographic examinations. In this study, high-resolution microcomputed tomography (*μ*CT40; Scanco Medical, Bassersdorf, Switzerland) was used to scan and characterize the bone profile regionally, which was presented by the morphometric indices computed on three bone levels of femur and tibia: whole bone, cortical bone in diaphysis, and trabecular bone in metaphysis. The bone samples were placed in a 12.3 mm diameter sample holder in 70% ethanol and immobilized with plastic foams. The samples were scanned at 8 *μ*m resolution. Morphometric and architectural parameters of bones were assessed and realistic 3D visual models were constructed for the object by selecting the volumes of interest (VOI). Data were acquired at an energy level of 55 keV, with 2000 projections, an integration time 300 ms, and an intensity of 109 *μ*A. 3D trabecular parameters were evaluated using a fixed Gaussian filter and a threshold of 220 for cancellous bone and 250 for cortical envelope.

#### 2.3.1. Whole Bone Analysis

For whole bone analysis, three parameters were measured: length, mineralized volume, and material bone mineral density. Sometimes engineers referred material bone mineral density (mBMD) to tissue mineral density (TMD), as a comparison to bone mineral density (BMD).

#### 2.3.2. Cortical Bone Analysis

For cortical bone analysis, a cross-sectional region of 100 transverse slices (a total length of 0.8 mm) at the middle of the bone was acquired. For each measurement point acquired at the same settings as the trabecular site, cortical area (Ct. Ar.), cross-sectional or total area (CSA), marrow area (Ma. Ar), and cortical thickness (Ct. Th.) were evaluated with the same Gaussian filter on a 0.5 mm region (50 slices). Area moment of inertia (*I*_min⁡_ and *I*_max⁡_) was evaluated on the same region.

#### 2.3.3. Cancellous Bone Analysis

For trabecular bone analysis, a region of 100 transverse slices at the secondary spongiosa in distal femur or distal tibia site was measured (Figure 3). The bone volume fraction was calculated directly by plotting gray voxels representing bone fraction against gray plus black voxel (nonbone objects; VOX BV/TV). Bone surface (BS) was calculated using a tetrahedron meshing technique generated by the “marching cubes method” and total volume (TV) was taken as the volume of interest (VOI). The normalized indices (BV/TV, BS/BV, and BS/BV) were used [8].

3D metric indices were calculated using direct techniques based on the distance transformation, without assuming a constant model. Direct indices Tb. Th, Tb. Sp, and Tb. N were calculated following distant transformation method.

The plate-rod characteristic of the structure was estimated by the structure model index (SMI). The geometric degree of anisotropy (DA) is defined as the ratio between the maximal and minimal radius of the MIL ellipsoid. Connectivity density (Conn. Dens.) was calculated using the Euler method of Odgaard and Gundersen [9].

### 2.4. Statistical Considerations

Mixed effects models were constructed to evaluate the association of strains with various bone properties, adjusting for the effects of gender and age, where each strain was considered to be an independent cluster and measurements from mice within a strain were considered to be repeated measurements for that cluster. Association between femur and tibia in terms of a given bone property of interest was described using Spearman's rank correlation coefficient for each strain with at least 4 paired measurements. *P* values are not adjusted for multiplicity and the results must be considered in the context of hypothesis generation. All analyses were conducted on SAS 9.3 (SAS Institute, Cary, NC).

## 3. Results

### 3.1. Significant Differences Were Observed among Bone Morphologies Strainwise

Mouse strain was significantly associated with all measured phenotypes after adjusting for gender and age except for cortical bone volume fraction (Table 1). That is, the variation of quantitative bone features due to genetic variation would still be significant when both age and gender were included in the model.

Our data shows that gender appeared to have a significant impact over most phenotypes as well (Table 1). It meant that bone features of female and male appear to be largely different in the sample space. The direction of the influence was denoted in the parenthesis with “F−” representing female values smaller than male values and “F+” indicating larger values in the females. Tibia and femur tended to be impacted by gender in the same orientation. For example, in mineralized volume, females tend to have smaller values than males in both femur and tibia (*P* < 0.0001). However, it was not always true that quantitative phenotypes of long bones were smaller in females. For example, females are inclined to have denser femur (*P* = 0.51) than males. Also, the females presented higher trabecular SMI than males which indicated more plate-like trabecular structures in female mice than males. These differences were therefore indicative of genetically based influence.

Our data also indicate that most phenotypes did not show significant difference due to age disparity. Therefore, age disparity appeared to not influence the data much, and the age difference in sampling did not show a significant difference.

### 3.2. Correlation of Bone Morphology between Femur and Tibia across Strains

The existence of rank correlations varied from strain to strain. In whole bone profile, 16 strains showed strong association in cortical thickness between femur and tibia (*P* < 0.05), while 2 were found with significant association in the BMD at distal femur (Supplementary Table S1 through Table S3 in Supplementary Material available online at http://dx.doi.org/10.1155/2015/728278). In the correlation of bone mineralized volume, BXD44 showed a perfect rank correlation between femur and tibia (*P* < 0.0001) while BXD95 was also observed to present the same correlation (*P* < 0.0001); however, in the measurement of mBMD, BXD44 was examined to reveal a negative correlation between femur and tibia (*r* = −0.4, *P* = 0.6) while BXD95 presented an association of 0.9 (*P* = 0.04).

**Figure 1:**
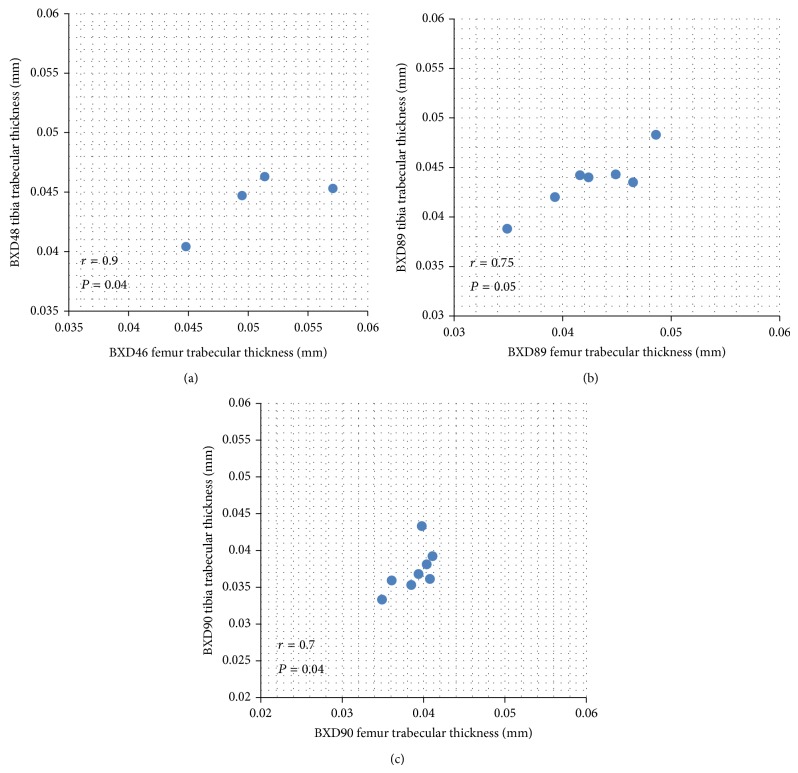
Spearman's rank correlations in trabecular thickness showed strong positive association between femur and tibia. (a) RI Strain BXD48 (*r* = 0.9, *P* = 0.04); (b) RI Strain BXD89 (*r* = 0.75, *P* = 0.05); (c) RI Strain BXD90 (*r* = 0.7, *P* = 0.04).

In addition, the degrees of correlations between femur and tibia vary across strains. We observed positive correlations from 0.7 to 1 (in trabecular envelope) and negative correlations from −0.86 to −1 (in whole bone envelope). Even within the same bone phenotype, a variety of correlation coefficients were found across strains. For example, in trabecular thickness, BXD89 and BXD90 revealed significant correlations at 0.75 (*P* = 0.05) and 0.7 (*P* = 0.04), respectively, while BXD48 showed a significant correlation of 0.9 (*P* = 0.04) as seen in Figure 1. This result showed that femur and tibia bone properties could relate to a various extent.

**Figure 2:**
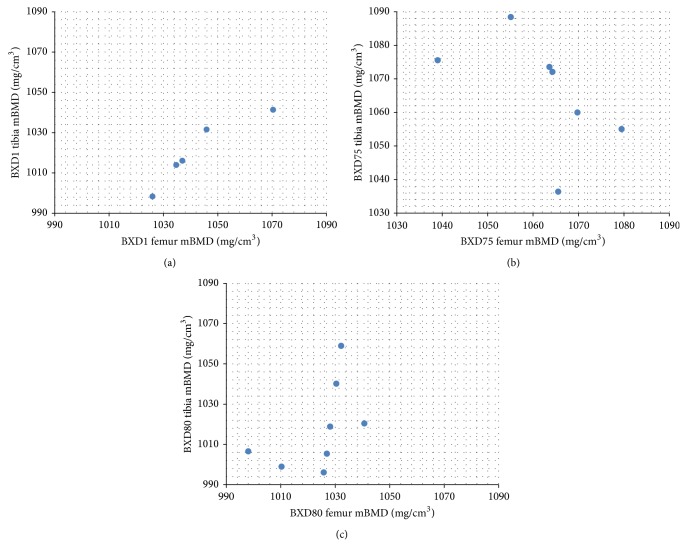
Spearman's rank correlations in mBMD differed between strains. (a) RI Strain BXD1 (*r* = 1.0, *P* < 0.0001); (b) RI Strain BXD75 (*r* = −0.86, *P* = 0.01); (c) RI Strain BXD80 (*r* = 0.9, *P* = 0.04).

Moreover, there was a combination of positive and negative correlations observed in phenotypes across strains. The sign of the correlation coefficient (+ or −) represented the direction of association between femur and tibia. In positive relationships, the increasing of femoral values' ranks would be accompanied by ascending tibial phenotypic ranks; in negative relationships, the tibial phenotypes presented a reverse rank order when femoral features were ascending among subjects. For example, BXD1 and BXD80 mice with higher mineral density in femur tended to have denser tibias (*r* = 1.0, *P* < 0.0001, and *r* = 0.76, *P* = 0.03). However, BXD75 mice's tibias were less dense in those with higher mineral density in femur (*r* = −0.86, *P* = 0.01) as shown in Figure 2.

### 3.3. Correlation of Bone Morphology between Femur and Tibia within Strains

**Figure 3:**
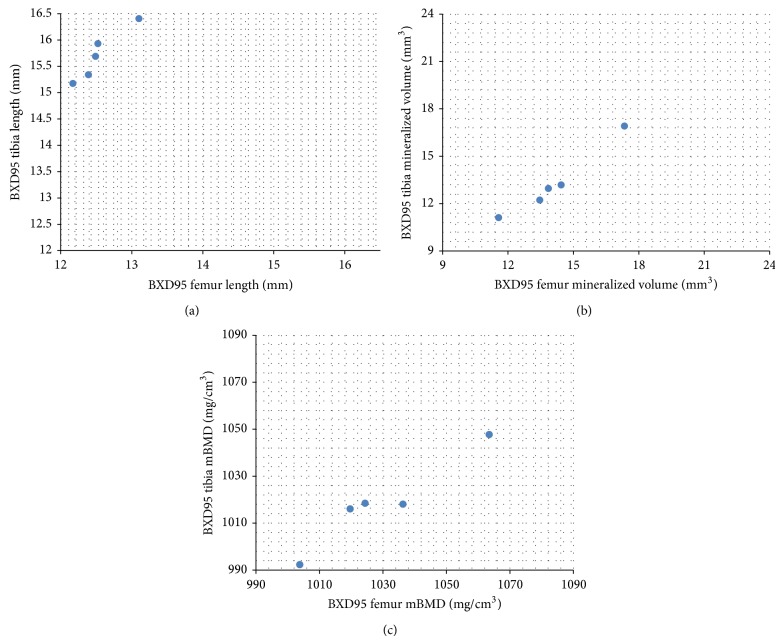
Spearman's rank correlations in RI Strain BXD95 whole bone profile showed a variety of associations between femur and tibia. (a) *r* = 1.0, *P* < 0.0001; (b) *r* = 1.0, *P* < 0.0001; (c) *r* = 0.9, *P* = 0.04.

Variations in correlations were found not only across strains but also within strains. First of all, correlations were found in various combinations of phenotypes strainwise. In whole bone profile, some strains showed significant femur-tibia correlation in all three measured phenotypes, including length, mineralized volume, and material bone mineral density (Figure 3); some strains showed significant long bone associations in two phenotypes (e.g., BXD60 and BXD62) and some revealed strong relationships in only one phenotype (e.g., BXD80). In trabecular envelope, multiple phenotypes in most strains showed significant correlations between femur and tibia. For example, BXD89 revealed significant correlation between femur and tibia in trabecular bone volume fraction (*ρ* = 0.89, *P* = 0.01), trabecular connectivity density (*ρ* = 0.96, *P* < 0.001), trabecular SMI (*ρ* = 0.86, *P* = 0.01), trabecular thickness (*ρ* = 0.75, *P* = 0.05), trabecular number (*ρ* = 0.93, *P* < 0.05), and trabecular space (*ρ* = 0.93, *P* < 0.05).

Secondly, a variety of correlations were observed within the same strains. Some bone features presented perfect ranking correlation (e.g., *r* = 1) while some showed a smaller correlation. Take BXD89's trabecular profile for an example; femur-tibia correlation was found at 0.89 in trabecular bone volume ratio (*P* = 0.01) while a stronger correlation (*r* = 0.96, *P* < 0.001) was discovered in the connectivity density. Similarly, different correlations were revealed in independent parameters, such as SMI (*r* = 0.86, *P* = 0.01), thickness (*r* = 0.75, *P* = 0.05), and trabecular number (*r* = 0.93, *P* < 0.05).

Finally, in the same strain of mice, femur and tibia correlated in different directions indicated by the positive and negative associations derived across morphological parameters. For example, in both strain BXD1 and strain BXD100, femur and tibia correlated in a negative direction in cross-sectional area of the cortical bone (*r* = −0.90, *P* = 0.04, and *r* = −0.89, *P* = 0.02), while they were revealed with positive relationship in cortical bone area (*r* = 0.9, *P* = 0.04, and *r* = 0.93, *P* = 0.01). In another, in these two strains of mice, femurs with wider midshafts jointed with tibias with narrower midshaft, even though mice with thicker cortical bone in femur carried tibias with similar features.

## 4. Discussion

While positive correlations between femur and tibia bone properties exist in a majority of strains, few strains showed a negative correlation. Although it is not clear how many of such cases exist in human population, our results seem to suggest that, in some cases, the bone properties in one part of the body may not represent the properties of the whole body or other parts. Because these genetically distinct RI strains were raised in a controlled environment (i.e., nutritional intake, physical activity, etc.); the differences observed in skeletal traits were the result of genetic variation.

In our study, the CV for femur midshaft area and cortical bone cross-sectional area is 22.8% and 16.6% which is consistent with a high degree of genetically determined variation in midshaft geometry reported by [3]. The data presented in this paper show remarkable differences in cross-sectional area of cortical bone and its mineral component, bone area, which is consistent with findings from Jepsen's group [10]. The differences of tibial geometry (i.e., curvature) would largely contribute to the mechanical strength testing and result in inconsistence and substantial errors. The considerable variation in bone intrinsic bone strength is consistent with the large variation in extrinsic bone strength from Wergedal et al. [3].

As stated in Statistical Considerations section, *P* values for the rank correlations in Table 1 are not adjusted for multiple testing; therefore, the significant findings at the traditional level of 0.05 must be considered as suggestive indications of associations to generate hypotheses to be tested in larger independent studies. Similarly, the lack of significant associations should be considered in a similar context as the lack of significance may be due to the small sample size (i.e., lack of power), not necessarily due to the inexistence of such associations.

Bone quantity and architecture are regulated genetically across skeletal sites. Despite the variation of trabecular bone within femur at different anatomical sites [11], the trabecular architecture showed close association between metaphyseal femur and metaphyseal tibia. This correlation may indicate that there are common genes in regulating lower limb development or that the development of specific skeletal sites on femur resembles that on tibia.

This strain also appeared to be different from other strains in tibial strain-specific parameters. It is possible that BXD98 is in possession of pleiotropic quantitative trait loci (QTLs). Further study coupled with linkage analysis is required to identify such QTL.

In this study, we employed the micro-CT technology for the measurement of several bone properties. Over the past decade, micro-CT has become an established imaging method in the study of variety of bone properties using mouse model [12]. The technology has the key advantage of performing nondestructive imaging of an object in three dimensions [13]. The micro-CT image can precisely determine the bone cross-sectional geometry and other mechanical properties. However, because the image is based on the X-ray signal of mineral contents, the properties measured by micro-CT do not represent the whole mechanic strength of the bone [14]. Soft tissues such as cartilage are generally undetectable by micro-CT due to their low X-ray attenuation. Therefore, we want to make readers aware of these facts in terms of interpreting our data.

In this study, we analyzed the genetic effect of bone properties between femur and tibia by the correlationship of these properties. Because the mouse strains in the study are homozygous RI strains derived from the same two progenitors, we assumed that the correlation reflects their true genetic relationship. Thus, if the correlation is a negative value between two traits, we then think that they are genetically negatively influencing each other. If the data of an analysis between two traits is a positive value, we then believe these two traits are genetically positively affecting each other. Unlike the traditional analysis from a generation to the next generation, this is a population based test. It is important to test the data in the separate independent studies to confirm some important findings in our study.

## Supplementary Material

Supplementary materials (Table S1-S3) provide information of the correlations among different bone properties between the female and the male and between the femur and the tibia. Table S1. Spearman's rank correlation coefficients and significance level of whole bone phenotypes between femur and tibia in RI mice (N>2). *P* values less than 0.05 and their variable values are highlighted with light grey. Only strains with mouse number of 3 or more are used for the analysis. Table S2. Spearman's rank correlation coefficients and significance level of cortical bone phenotypes between femur and tibia in RI mice (N>2). P values less than 0.05 and their variable values are highlighted with light grey. Table S3. Spearman's rank correlation coefficients and significance level of cortical bone phenotypes between femur and tibia in RI mice (N>2). *P* values less than 0.05 and their variable values are highlighted with light grey. Trab BV/TV = Trabecular bone volume density, or bone volume ratio, Trab Conn. Dens = Trabecular connectivity density, Trab SMI = Trabecular structural Model Index, Trab Th = Trabecular thickness, Trab N= Trabecular number, Trab Sp = Trabecular space, Trab mBMD = Material bone mineral density.

## Figures and Tables

**Table 1:** *P* value of strain effect on bone morphological and biomechanical properties in femur and tibia of RI mice adjusted for gender and age (*N* > 3).

Measurement^*^	Bone	Number of strains	Percentage of females	Age range (week)	Strain	Gender	Age
Length	Femur	46	48.9	9.6–13.6	*<0.0001 *	*<0.0001 (F−) *	*0.04 (+) *
	Tibia	46	52.0	9.6–13.6	*<0.0001 *	**0.075 (F−)**	0.23

mBMD	Femur	46	48.9	9.6–13.6	*<0.0001 *	**0.051 (F+)**	0.8
	Tibia	46	52.0	9.6–13.6	*<0.0001 *	0.53	**0.096 (+)**

Min. Vol	Femur	46	49.5	9.6–13.6	*<0.0001 *	*<0.0001 (F−) *	*0.039 (+) *
	Tibia	46	52.0	9.6–13.6	*<0.0001 *	*<0.0001 (F−) *	0.35

Ct. Th	Femur	46	55.8	9.6–13.6	*<0.0001 *	*<0.0001 (F−) *	0.22
	Tibia	46	54.5	9.6–13.6	*<0.0001 *	*<0.0001 (F−) *	0.18

Ct. mBMD	Femur	46	55.8	9.6–13.6	*<0.0001 *	*0.014 (F−) *	0.86
	Tibia	46	54.5	9.6–13.6	*<0.0001 *	*0.02 (F−) *	**0.09 (+)**

CSA	Femur	46	55.8	9.6–13.6	*0.0048 *	*<0.0001 (F−) *	0.59
	Tibia	46	54.5	9.6–13.6	*0.01 *	*0.0001 (F−) *	0.21

Ct. Ar	Femur	46	55.8	9.6–13.6	*<0.0001 *	*<0.0001 (F−) *	0.82
	Tibia	46	54.5	9.6–13.6	*<0.0001 *	*<0.0001 (F−) *	0.43

Trab. BV/TV	Femur	46	53.9	9.6–13.6	*<0.0001 *	*<0.0001 (F−) *	0.4
	Tibia	46	53.7	9.7–13.6	*<0.0001 *	*<0.0001 (F−) *	0.8

Conn. Dens	Femur	46	53.9	9.6–13.6	*<0.0001 *	*<0.0001 (F−) *	**0.05 (−)**
	Tibia	46	53.7	9.7–13.6	*<0.0001 *	*<0.0001 (F−) *	**0.087 (−)**

Trab. SMI	Femur	46	53.8	9.6–13.6	*<0.0001 *	*<0.0001 (F+) *	0.48
	Tibia	46	53.7	9.7–13.6	*<0.0001 *	*<0.0001 (F+) *	0.81

Trab. N	Femur	46	53.9	9.6–13.6	*<0.0001 *	*<0.0001 (F−) *	*0.017 (−) *
	Tibia	46	53.7	9.7–13.6	*<0.0001 *	*<0.0001 (F−) *	0.25

Trab. Th	Femur	46	53.9	9.6–13.6	*<0.0001 *	*<0.0001 (F−) *	0.87
	Tibia	46	53.7	9.7–13.6	*<0.0001 *	*<0.0001 (F−) *	*0.01 (+) *

Trab. Sp	Femur	46	53.9	9.6–13.6	*<0.0001 *	*<0.0001 (F+) *	*0.009 (+) *
	Tibia	46	53.7	9.7–13.6	*<0.0001 *	*<0.0001 (F+) *	0.35

Trab. mBMD	Femur	46	53.9	9.6–13.6	*<0.0001 *	*0.0007 (F−) *	0.23
	Tibia	46	53.7	9.7–13.6	*<0.0001 *	*0.018 (F−) *	0.65

Trab. DA	Femur	46	53.9	9.6–13.6	*<0.0001 *	0.15	**0.1 (−)**
	Tibia	46	53.7	9.7–13.6	*<0.0001 *	**0.08 (F−)**	0.91

^*^ *P* values no greater than 0.05 are in italic and they are in bold if they were larger than 0.05 but less than 0.1. F+ means females have higher values; F− means females have lower values. (−) refers to negative association; (+) refers to positive association. Min. Vol = mineral volume, Ct. Th = cortical thickness (mm), CSA = cross-sectional area (mm^2^), Ct. Ar = cross-sectional area of cortical bone (mm^2^), Trab. BV/TV = trabecular bone volume density or bone volume ratio, Conn. Dens = connectivity density, Trab. SMI = trabecular structural model index, Trab. N = trabecular number, Trab. Th = trabecular thickness, Trab. Sp = trabecular space, Trab. mBMD = trabecular material bone mineral density, and Trab. DA = trabecular degree of anisotropy.

## Appendix

### Protocol for Harvesting Mouse Femur and Tibia

*Materials and Reagents*

Inbred mice,
CO_2_,
70% ethanol,
1.5 mL tubes,
sharp dissect scissor (sterile or sprayed with 70% ethanol),
blunt end scissor (sterile or sprayed with 70% ethanol).

*Procedure*

1. Immediately before surgery, sacrifice mice with CO_2_ asphyxiation followed by cervical dislocation.
2. Mice were then put on dissection board and sprayed with 70% ethanol.
3. Remove fur and skin from legs by lifting skin at the base of each leg with tweezers and cutting away skin across thigh and down to ankle.
4. Using blunt end scissors, make an incision 1 inch vertically from umbilical region to anterior region.
5. Extend this incision along the medial aspect of both rear appendages.
6. Peel skin down leg and over foot and firmly tug until it is removed.
7. Use sharp scissors to remove muscle from entire leg so that bone is completely exposed.
8. Clean bones of any remaining muscle and place femur and tibia in 1.5 mL tubes, respectively, containing 70% EtOH.
9. Store tubes with specimens in room temperature for experiments.
