# Supplementary material for: Bone Morphology in 46 BXD Recombinant Inbred Strains and Femur-Tibia Correlation
Source: ScientificWorldJournal. 2015 Feb 25;2015:728278. doi: 10.1155/2015/728278 (PMC4355808; doi:10.1155/2015/728278)
Supplement: Supplementary file 1 — Supplementary materials (Table S1-S3) provide information of the correlations among different bone properties between the female and the male and between the femur and the tibia. Table S1. Spearman's rank correlation coefficients and significance level of whole bone phenotypes between femur and tibia in RI mice (N>2). P values less than 0.05 and their variable values are highlighted with light grey. Only strains with mouse number of 3 or more are used for the analysis. Table S2. Spearman's rank correlation coefficients and significance level of cortical bone phenotypes between femur and tibia in RI mice (N>2). P values less than 0.05 and their variable values are highlighted with light grey. Table S3. Spearman's rank correlation coefficients and significance level of cortical bone phenotypes between femur and tibia in RI mice (N>2). P values less than 0.05 and their variable values are highlighted with light grey. Trab BV/TV = Trabecular bone volume density, or bone volume ratio, Trab Conn. Dens = Trabecular connectivity density, Trab SMI = Trabecular structural Model Index, Trab Th = Trabecular thickness, Trab N= Trabecular number, Trab Sp = Trabecular space, Trab mBMD = Material bone mineral density. [file 728278.f1.pdf]

APPENDIX B. SUPPOTIVE TABLES FOR DATA ANALYSIS

Table S1. Spearman's rank correlation coefficients and significance level of whole bone phenotypes between femur and tibia in RI mice (N>2).

Strain	N	Length	Length (P-value)	Mineralized Volume	Mineralized Volume (P-value)	mBMD	mBMD (P-value)
**B6D2**	6	0.60	0.21	0.83	0.04	-0.09	0.87
**BXD1**	5	0.90	0.04	1.00	<0.0001	1.00	<0.0001
**BXD100**	7	-0.29	0.54	-0.11	0.82	0.25	0.59
**BXD101**	3	0.50	0.67	0.50	0.67	1.00	<0.0001
**BXD102**	6	-0.49	0.33	0.14	0.79	0.38	0.46
**BXD103**	2	NA	NA	NA	NA	NA	NA
**BXD11**	3	0.50	0.67	0.50	0.67	1.00	<0.0001
**BXD12**	5	-0.40	0.50	-0.10	0.50	0.30	0.62
**BXD14**	4	-0.60	0.40	0.20	0.80	0.80	0.20
**BXD24**	3	1.00	<0.0001	1.00	<0.0001	1.00	<0.0001
**BXD27**	5	0.10	0.87	0.80	0.10	-0.10	0.87
**BXD29**	4	0.40	0.60	0.80	0.20	0.60	0.40
**BXD31**	2	NA	NA	NA	NA	NA	NA
**BXD32**	7	-0.36	0.43	0.32	0.48	0.54	0.22
**BXD34**	3	0.50	0.67	0.50	0.67	-1.00	<0.0001
**BXD38**	2	NA	NA	NA	NA	NA	NA
**BXD42**	3	1.00	<0.0001	0.50	0.67	0.50	0.67
**BXD44**	4	1.00	<0.0001	1.00	<0.0001	-0.40	0.60
**BXD45**	2	NA	NA	NA	NA	NA	NA
**BXD48**	4	0.80	0.20	0.40	0.60	0.80	0.20

Table S1. (Cont'd).

Strain	N	Length	Length (P-value)	Mineralized Volume	Mineralized Volume (P-value)	mBMD	mBMD (P-value)
**BXD49**	4	0.80	0.20	0.80	0.20	-0.63	0.37
**BXD50**	5	0.90	0.04	1.00	<0.0001	0.60	0.29
**BXD56**	4	0.80	0.20	0.80	0.20	-0.40	0.60
**BXD60**	7	0.93	<0.01	1.00	<0.0001	0.46	0.29
**BXD62**	8	0.91	<0.01	0.83	0.01	0.48	0.23
**BXD63**	3	1.00	<0.0001	1.00	<0.0001	0.50	0.67
**BXD68**	4	1.00	<0.0001	1.00	<0.0001	-0.40	0.60
**BXD69**	4	0.80	0.20	0.80	0.20	0.40	0.60
**BXD70**	7	0.21	0.65	0.61	0.15	0.25	0.59
**BXD71**	6	-0.43	0.40	0.37	0.47	0.31	0.54
**BXD73**	2	NA	NA	NA	NA	NA	NA
**BXD75**	7	-0.21	0.65	-0.11	0.82	-0.86	0.01
**BXD79**	6	0.94	<0.01	0.94	<0.01	-0.43	0.40
**BXD80**	8	-0.16	0.71	0.60	0.12	0.76	0.03
**BXD83**	6	0.37	0.47	0.77	0.07	-0.66	0.16
**BXD84**	5	-0.80	0.10	-0.40	0.50	0.30	0.62
**BXD85**	3	0.50	0.67	1.00	<0.0001	1.00	<0.0001
**BXD87**	3	-1.00	<0.0001	0.50	0.67	-0.50	0.67
**BXD89**	6	0.71	0.11	0.94	<0.01	0.26	0.62
**BXD90**	9	0.90	<.001	0.78	0.01	0.65	0.06
**BXD92A**	5	0.30	0.62	0.50	0.39	0.40	0.50
**BXD95**	5	1.00	<0.0001	1.00	<0.0001	0.90	0.04
**BXD97**	4	0.40	0.60	-0.20	0.80	0.40	0.60

Table S1. (Cont'd).

Strain	N	Length	Length (P-value)	Mineralized Volume	Mineralized Volume (P-value)	mBMD	mBMD (P-value)
**C57BL/6J**	13	0.08	0.79	0.04	0.89	-0.13	0.67
**D2B6F1**	6	0.26	0.62	0.60	0.21	-0.26	0.62
**DBA2J**	10	-0.36	0.30	-0.49	0.15	-0.26	0.47

P values less than 0.05 and their variable values are highlighted with light grey. Only strains with mouse number of 3 or more are used for the analysis.

Table S2. Spearman's rank correlation coefficients and significance level of cortical bone phenotypes between femur and tibia in RI mice (N>2).

Strain	N	Ct. mBMD	Ct. mBMD (P-value)	Ct. Th	Ct. Th (P-value)	CSA	CSA (P-value)	Ct. Ar	Ct Ar (P-value)
**B6D2**	8	0.29	0.49	0.79	0.02	0.32	0.43	0.57	0.14
**BXD1**	5	-0.50	0.39	0.40	0.50	-0.90	0.04	0.90	0.04
**BXD100**	6	0.43	0.40	0.67	0.16	-0.89	0.02	0.93	0.01
**BXD101**	6	-0.60	0.21	-0.26	0.62	0.54	0.27	0.23	0.66
**BXD102**	6	-0.26	0.62	0.20	0.70	0.83	0.04	0.14	0.79
**BXD103**	3	0.50	0.67	0.50	0.67	0.50	0.67	0.50	0.67
**BXD11**	4	0.40	0.60	1.00	<0.0001	0.60	0.40	0.60	0.40
**BXD12**	5	-0.50	0.39	0.60	0.28	-0.30	0.62	0.10	0.87
**BXD14**	5	-0.80	0.10	0.00	1.00	-0.30	0.62	0.70	0.19
**BXD24**	5	-0.90	0.04	-0.60	0.28	0.30	0.62	0.70	0.19
**BXD27**	4	0.20	0.80	0.80	0.20	-1.00	<0.0001	1.00	<.0001
**BXD29**	4	-0.20	0.80	1.00	<0.0001	0.80	0.20	0.40	0.60
**BXD31**	2	NA	NA	NA	NA	NA	NA	NA	NA
**BXD32**	6	0.49	0.33	0.94	<0.01	0.14	0.79	0.46	0.35
**BXD34**	1	NA	NA	NA	NA	NA	NA	NA	NA
**BXD38**	4	0.40	0.60	-0.80	0.20	-1.00	<0.0001	0.20	0.80
**BXD42**	4	0.40	0.60	1.00	<0.0001	-1.00	<0.0001	-0.63	0.37
**BXD44**	7	0.04	0.94	0.89	0.01	0.68	0.09	0.82	0.02
**BXD45**	3	-0.50	0.67	1.00	<0.0001	-0.50	0.67	-0.50	0.67
**BXD48**	7	0.32	0.48	0.96	<0.001	0.80	0.02	0.89	0.01
**BXD49**	7	-0.50	0.25	0.75	0.05	0.71	0.07	0.96	<0.001
**BXD50**	5	0.50	0.39	1.00	<0.0001	-0.20	0.75	1.00	<0.0001

Table S2. (Cont'd).

Strain	N	Ct. mBMD	Ct. mBMD (P-value)	Ct. Th	Ct. Th (P-value)	CSA	CSA (P-value)	Ct. Ar	Ct Ar (P-value)
**BXD56**	5	0.40	0.50	0.90	0.04	0.90	0.04	0.82	0.09
**BXD60**	6	0.14	0.79	1.00	<0.0001	0.54	0.27	0.94	<0.01
**BXD62**	9	0.33	0.38	0.83	0.01	0.23	0.56	0.87	<0.01
**BXD63**	4	0.00	1.00	0.80	0.20	-0.20	0.80	0.40	0.60
**BXD68**	4	-0.32	0.68	-0.60	0.37	0.00	1.00	0.10	0.89
**BXD69**	6	-0.03	0.96	0.83	0.04	0.70	0.11	0.49	0.33
**BXD70**	7	0.46	0.29	0.60	0.15	-0.07	0.88	0.68	0.09
**BXD71**	7	-0.66	0.16	0.54	0.27	0.09	0.87	0.94	0.00
**BXD73**	5	1.00	<0.0001	0.00	1.00	0.00	1.00	0.00	1.00
**BXD75**	7	-0.29	0.53	0.64	0.12	-0.04	0.94	0.96	<0.001
**BXD79**	6	-0.54	0.27	0.94	<0.01	0.70	0.11	1.00	<0.0001
**BXD80**	9	-0.05	0.90	0.37	0.33	-0.55	0.13	0.47	0.21
**BXD83**	7	-0.36	0.43	0.25	0.59	0.27	0.55	0.46	0.29
**BXD84**	5	0.80	0.10	0.00	1.00	-0.30	0.62	-0.10	0.87
**BXD85**	3	1.00	<0.0001	1.00	<0.0001	0.50	0.67	1.00	<.0001
**BXD87**	5	0.90	0.04	0.60	0.28	0.70	0.19	0.70	0.19
**BXD89**	5	0.70	0.19	0.80	0.10	0.50	0.39	0.90	0.04
**BXD90**	9	0.37	0.33	0.82	0.01	0.43	0.24	0.83	0.01
**BXD92A**	5	-0.50	0.39	-0.10	0.87	-0.10	0.87	0.50	0.39
**BXD95**	5	0.90	0.04	0.70	0.19	0.70	0.19	0.70	0.19
**BXD97**	4	0.80	0.20	1.00	<0.0001	0.32	0.68	0.80	0.20
**C57BL/6J**	12	0.12	0.70	0.29	0.37	0.20	0.50	0.40	0.15
**D2B6F1**	6	0.14	0.79	0.94	<0.01	0.77	0.07	0.94	<0.01
**DBA2J**	11	-0.01	0.98	-0.06	0.85	-0.28	0.41	-0.18	0.59

P values less than 0.05 and their variable values are highlighted with light grey.

Table S3. Spearman's rank correlation coefficients and significance level of cortical bone phenotypes between femur and tibia in RI mice (N>2).

Strain	N	Trab BV/TV	Trab BV/TV (P-value)	Trab Conn.Dens	Trab Conn.Dens (P-value)	Trab SMI	Trab SMI (P-value)	Trab Th	Trab Th (P-value)	Trab N	Trab N (P-value)	Trab Sp	Trab Sp (P-value)	Trab mBMD	Trab mBMD (P-value)
**BXD1**	4	0.80	0.20	0.20	0.80	1.00	<0.0001	0.80	0.20	0.20	0.80	0.20	0.80	0.80	0.20
**BXD38**	4	0.40	0.60	-0.40	0.60	0.40	0.60	1.00	<0.0001	-0.20	0.80	-0.20	0.80	0.80	0.20
**BXD45**	4	0.80	0.20	1.00	<0.0001	0.40	0.60	0.80	0.20	1.00	<0.0001	0.80	0.20	0.80	0.20
**BXD69**	4	1.00	<0.0001	0.80	0.20	1.00	<0.0001	0.80	0.20	0.60	0.40	0.80	2.00	0.40	0.60
**BXD73**	4	0.40	0.60	0.40	0.60	0.00	1.00	0.20	0.80	0.20	0.80	0.40	0.60	1.00	<0.0001
**BXD84**	4	-0.20	0.80	0.20	0.80	-0.40	0.60	0.60	0.40	-0.40	0.60	-0.40	0.60	0.40	0.60
**BXD95**	4	1.00	<0.0001	0.80	0.20	0.80	0.20	0.80	0.20	0.80	0.20	1.00	<0.0001	0.40	0.60
**BXD97**	4	0.40	0.60	0.40	0.60	-0.40	0.60	0.40	0.60	0.40	0.60	0.40	0.60	0.40	0.60
**B6D2**	5	0.60	0.28	0.30	0.62	0.50	0.39	0.80	0.10	0.60	0.28	0.50	0.39	0.60	0.28
**BXD101**	5	-0.10	0.87	0.60	0.28	-0.80	0.10	0.50	0.39	0.70	0.19	0.60	0.28	-0.80	0.10
**BXD27**	5	0.90	0.04	0.80	0.10	0.70	0.19	0.70	0.19	0.70	0.19	0.70	0.19	-0.40	0.50
**BXD48**	5	0.60	0.28	0.00	1.00	-0.50	0.39	0.90	0.04	0.00	1.00	0.00	1.00	-0.30	0.60
**BXD49**	5	0.90	0.04	1.00	<0.0001	0.90	0.04	0.30	0.62	1.00	<0.0001	1.00	<0.0001	0.40	0.50
**BXD92A**	5	0.05	0.90	-0.30	0.62	0.00	1.00	0.30	0.62	0.90	0.04	0.90	0.04	0.60	0.28
**D2B6F1**	5	0.50	0.39	0.60	0.62	0.50	0.39	0.00	1.00	0.20	0.74	0.20	0.75	0.60	0.28
**BXD100**	6	0.83	0.04	0.30	0.54	0.20	0.70	0.54	0.27	0.94	<0.05	0.89	0.02	0.14	0.79
**BXD12**	6	0.60	0.21	0.66	0.16	0.50	0.27	0.67	0.15	0.83	0.04	0.83	0.04	0.20	0.70

**BXD62**	6	0.71	0.11	0.89	0.02	0.83	0.04	0.03	0.96	0.83	0.04	0.77	0.07	0.60	0.21
**BXD71**	6	0.94	<0.05	0.43	0.40	0.54	0.27	-0.26	0.62	0.70	0.11	0.94	<0.01	0.60	0.21
**BXD79**	6	0.54	0.27	0.43	0.40	0.14	0.79	0.26	0.62	0.94	<0.05	0.89	0.02	0.66	0.16
**BXD80**	6	0.54	0.27	0.89	0.02	0.77	0.07	0.09	0.87	0.83	0.04	0.84	0.04	0.37	0.47
**BXD102**	7	-0.20	0.67	0.36	0.40	-0.07	0.88	-0.07	0.88	0.18	0.70	0.11	0.82	0.29	0.53
**BXD32**	7	0.82	0.02	0.43	0.34	0.89	0.01	0.54	0.22	0.54	0.22	0.46	0.29	-0.09	0.85

Table S3. (Cont'd).

Strain	N	Trab BV/TV	Trab BV/TV (P-value)	Trab Conn.Dens	Trab Conn.Dens (P-value)	Trab SMI	Trab SMI (P-value)	Trab Th	Trab Th (P-value)	Trab N	Trab N (P-value)	Trab Sp	Trab Sp (P-value)	Trab mBMD	Trab mBMD (P-value)
**BXD60**	7	0.79	0.04	0.64	0.12	0.86	0.01	0.74	0.06	0.64	0.12	0.64	0.12	0.79	0.04
**BXD70**	7	0.86	0.01	0.75	0.05	0.82	0.02	0.60	0.15	0.70	0.07	0.64	0.12	0.60	0.15
**BXD83**	7	0.36	0.43	0.68	0.09	0.57	0.18	0.07	0.88	0.68	0.09	0.68	0.09	0.25	0.59
**BXD89**	7	0.89	0.01	0.96	<0.001	0.86	0.01	0.75	0.05	0.93	<0.05	0.93	<0.05	0.00	1.00
**C57BL/6J**	8	0.36	0.39	0.17	0.69	-0.05	0.90	0.48	0.23	0.24	0.57	0.29	0.49	0.00	1.00
**DBA2J**	8	0.43	0.29	0.62	0.10	-0.17	0.69	0.31	0.45	0.52	0.18	0.64	0.09	0.24	0.57
**BXD90**	9	0.53	0.14	0.28	0.46	0.75	0.02	0.70	0.04	0.57	0.11	0.73	0.03	-0.20	0.59

P values less than 0.05 and their variable values are highlighted with light grey. Trab BV/TV = Trabecular bone volume density, or bone volume ratio, Trab Conn.Dens = Trabecular connectivity density, Trab SMI = Trabecular structural Model Index, Trab Th = Trabecular thickness, Trab N = Trabecular number, Trab Sp = Trabecular space, Trab mBMD = Material bone mineral density.
